# Smart Buildings: Water Leakage Detection Using TinyML

**DOI:** 10.3390/s23229210

**Published:** 2023-11-16

**Authors:** Othmane Atanane, Asmaa Mourhir, Nabil Benamar, Marco Zennaro

**Affiliations:** 1 School of Science and Engineering, Al Akhawayn University in Ifrane, P.O. Box 104, Hassan II Avenue, Ifrane 53000, Morocco; o.atanane@aui.ma (O.A.); n.benamar@aui.ma (N.B.); 2 School of Technology, Moulay Ismail University of Meknes, Meknes 50050, Morocco; 3 The Abdus Salam International Centre for Theoretical Physics, 34151 Trieste, Italy; mzennaro@ictp.it

**Keywords:** CNN, EfficientNet, TinyML, accelerometer, acoustic data, scalogram, deep learning

## Abstract

The escalating global water usage and the increasing strain on major cities due to water shortages highlights the critical need for efficient water management practices. In water-stressed regions worldwide, significant water wastage is primarily attributed to leakages, inefficient use, and aging infrastructure. Undetected water leakages in buildings’ pipelines contribute to the water waste problem. To address this issue, an effective water leak detection method is required. In this paper, we explore the application of edge computing in smart buildings to enhance water management. By integrating sensors and embedded Machine Learning models, known as TinyML, smart water management systems can collect real-time data, analyze it, and make accurate decisions for efficient water utilization. The transition to TinyML enables faster and more cost-effective local decision-making, reducing the dependence on centralized entities. In this work, we propose a solution that can be adapted for effective leakage detection in real-world scenarios with minimum human intervention using TinyML. We follow an approach that is similar to a typical machine learning lifecycle in production, spanning stages including data collection, training, hyperparameter tuning, offline evaluation and model optimization for on-device resource efficiency before deployment. In this work, we considered an existing water leakage acoustic dataset for polyvinyl chloride pipelines. To prepare the acoustic data for analysis, we performed preprocessing to transform it into scalograms. We devised a water leak detection method by applying transfer learning to five distinct Convolutional Neural Network (CNN) variants, which are namely EfficientNet, ResNet, AlexNet, MobileNet V1, and MobileNet V2. The CNN models were found to be able to detect leakages where a maximum testing accuracy, recall, precision, and F1 score of 97.45%, 98.57%, 96.70%, and 97.63%, respectively, were observed using the EfficientNet model. To enable seamless deployment on the Arduino Nano 33 BLE edge device, the EfficientNet model is compressed using quantization resulting in a low inference time of 1932 ms, a peak RAM usage of 255.3 kilobytes, and a flash usage requirement of merely 48.7 kilobytes.

## 1. Introduction

The presence of water is the distinguishing factor that allows Earth to harbor life. Recent studies show that global water usage is predicted to surge by 55% and almost a quarter of major cities worldwide are already grappling with some degree of water strain [1]. A lack of safe drinking water is one of the consequences of water shortages. Nearly 2.2 billion individuals globally are struggling to drink safe water [1].

In an attempt to deal with the alarming situation of water worldwide, different initiatives have been proposed and deployed. We can cite, for example, water restrictions, re-purposing water for non-potable use, and awareness campaigns.

However, one of the major problems faced by water management institutions worldwide is the high amounts of drinkable water that are wasted because of leaking pipes in the distribution networks [2]. Water wastage is caused by various factors, those that are related to human behavior, and those that are linked to the state and the quality of the pipes in distribution networks. Indeed, pipe flaws can lead to substantial losses of quality water and wasted energy in purification processes [3]. Morocco, representing a typical example from the Global South countries struggling with water scarcity, faces significant water wastage primarily due to leakages. According to a 2019 report by the World Bank, Morocco’s water supply experiences a loss of approximately 40% due to leaks, inefficient use and aging infrastructure [4]. This is a significant amount of water wastage that highlights the need for more efficient water management practices. Water leakages in buildings’ pipelines are a common and costly problem that can cause a wide range of issues. Leaks can be caused by a variety of factors such as age, wear and tear, corrosion, damage from external factors (temperature and humidity), poor installation, or the pipeline’s quality. They can occur in any part of the pipeline system, from the main water line to individual fixtures, and can result in significant water waste, increased water bills, structural damage, mold growth, and other related problems. The early detection and repair of leaks is crucial to minimize damage and avoid costly repairs.

Among the solutions to mitigate water stress is to improve water efficiency by reducing the quantity of non-revenue water. To this end, various techniques for leakage detection in buildings’ pipelines have been developed, ranging from visual inspection to advanced technologies such as cameras, acoustic, pressure, acceleration, and flow sensors. In this context, it is critical to identify the most effective and efficient methods to detect leaks.

In this work, we propose a solution for automatic water leakage detection to showcase a form of technical intelligence in smart buildings. The objective of intelligent buildings is to operate autonomously, possessing the ability to learn, forecast, and adapt without the need for user intervention or awareness. Sensors and monitors can rapidly and automatically adjust parameters such as room temperatures, lighting, shading, and the consumption of energy and water [5]. The application of information and communication technology, specifically through sensors and Machine Learning (ML) techniques, enhances the quality of life for occupants within these smart buildings, especially in urban settings. Smart cities should have buildings with smart water management technology to monitor, use and reuse water resources in a more efficient and sustainable way. At the heart of smart water management systems, we usually find technologies such as: (i) sensors to collect live data, with reporting capabilities to a master system, (ii) artificial intelligence and ML models to analyze vast amounts of data and identify patterns that would be difficult for human beings to determine easily, and (iii) intelligent controllers capable of making some local decision making.

Despite the improvements brought using traditional IoT systems and the different ML techniques, these systems suffer from serious drawbacks. Traditionally, IoT systems depended on offloading massive amounts of real-time sensor data to the cloud. Data is then processed to make required decisions, and reply to the appropriate nodes. However, this configuration faced major challenges, including high communication and processing loads as a significant delay which does not serve the needs of real time applications. Furthermore, data privacy problems developed when third-party companies became engaged in communication, storage, and decision-making processes. TinyML has emerged as a viable option to effectively address these challenges through the convergence of tiny intelligent devices and ML technologies [6,7]. TinyML is a specialized subset of edge computing [8,9] which enhances intelligence on resource-constrained edge devices, with limited processing power and memory, such as sensors and IoT objects. This enables devices to perform AI tasks locally, often with very low power consumption, without relying on cloud-based processing. As a result, the central entity’s work is drastically decreased or in certain circumstances abolished completely, with just periodic updates of metadata information required for supervision purposes.

This work delves deeper into smart water leak detection using TinyML, which would permit the refinement of water utilities by detecting leaks and tracking water distribution within a building.

Detecting water leaks with ML requires the usage of various sorts of data in order to effectively detect and locate probable leaks. Several data sources and approaches may be used in this process to detect and prevent leaks effectively [10]. Among these, sensors’ data play a pivotal role. Water distribution systems are equipped with numerous sensors that capture real-time data such as water flow rates, pressure levels, and vibrations. ML systems can discover abnormalities and trends that indicate anomalies by monitoring these metrics. A quick decrease in pressure or an unusual rise in flow rate, for instance, might indicate a possible leak. Acoustic data is another useful data source [10]. Water leaks frequently make distinct noises that may be detected by sophisticated acoustic sensors or even by analyzing audio recordings. ML algorithms that have been taught to recognize certain leak-related sound patterns can be used to detect leaks automatically. By leveraging these diverse data sources and employing ML algorithms, water utilities can improve their ability to detect leaks promptly.

The aim of our work is to develop TinyML models that are capable of detecting water leakage in pipes using acoustic data. In this study, we focus on deep learning models, especially Convolutional Neural Networks (CNNs). CNNs are employed to detect water leakages by utilizing the sensory input of a water distribution network. CNNs analyze the regional similarities in sensor inputs, detecting leaks by identifying deviations in sensor readings caused by changes in flow, pressure, or vibration. The CNN models effectively capture these differences and use them to pinpoint the presence of leaks within the water distribution network.

We also apply quantization on the different models to identify the best performing and efficient model for a low-cost device which is Arduino Nano 33 BLE (https://store.arduino.cc/products/arduino-nano-33-ble (accessed on 1 October 2023)).

The subsequent sections of this paper are structured as follows: Section 2 investigates the related work, presenting previous research addressing the same issue. In Section 3, we detail the materials and methods deployed for conducting our experiments. Section 4 presents the empirical results and discusses the outcomes derived from the experiments. Lastly, Section 5 highlights some conclusions and directions for future work.

## 2. Related Works

Numerous studies have tackled the use of machine learning or deep learning based CNN architectures to discern patterns and detect irregularities within water and wastewater pipeline systems.

For example, Fang et al. [11] present a CNN-based method for identifying numerous leakage spots. The CNN model collects important features from past leakage data and applies them to real-time data to detect whether there is a leak. A simulated water distribution system experimental platform was constructed within the AnhuWe Province Key Laboratory of Intelligent Building and Building Energy Saving. The platform spans 200 m^2^, with pipe sections measuring 400 m in length and featuring pipe diameters ranging from 30 cm to 50 cm. A total of 21 water pressure sensors were strategically placed throughout the platform. They were able to collect a dataset that contains non-leakage data and four types of pipe network pressure data under the conditions of single-point leakages, two-point leakages, and three-point leakages. The experimental findings show great detection accuracies, with 99.63%, 98.58%, and 95.25% accuracy attained for one, two, and three leakage spots employing 21 sensors, respectively. When the number of sensors is reduced to eight, the accuracies for one, two, and three leakage points drop to 96.43%, 94.88%, and 91.56%, respectively.

In Kang et al. [12], the authors conducted non-invasive measurement of the leakage signals using piezoelectric accelerometers (PCB-393B31). These accelerometers possess the ability to objectively measure vibrations and translate them into acceleration levels. Furthermore, the authors introduced a local search technique based on graphs to locate leaks. Their approach, however, was not completely tuned for categorizing 1-D signals. It required feature extraction from the recorded signal data prior to applying the classification layers. Furthermore, the detection range was determined by the clarity and correlation of acoustic signals, and the issue of mistakes caused by signals with low correlation coefficients remained unsolved.

In a different study by Cody et al. [13], the authors designed a water system experimental test bed that is made up of many components, including a full-scale hydrant and PVC pipes with bends. These pipes are made of grayscale schedule 80 PVC with a 152.4 mm inner diameter, which is extensively used in water distribution networks (WDNs) in Canada and the United States. The authors used a CNN architecture whose output is sent into a variational autoencoder (VAE), which tries to recover the original spectrogram image. The mean squared error (MSE) between the original image and its reconstructed counterpart is used to calculate the loss function. This proposed method achieved an accuracy of 97.2% for detecting a 0.25 L/s leak.

Shukla et al. [14] conducted a study where a CNN model is built using modified layers of the pre-trained AlexNet network [15]. The purpose of the model is to classify images based on different scenarios using a dataset of 9000 scalograms images. This was achieved by considering 25 scenarios, with 12 accelerometers per scenario, 10 samples per scenario, and three images per sample. The model excels at categorizing images based on their corresponding leakage scenarios, accurately identifying healthy configurations as well as various leaky situations with a 95% accuracy rate. Moreover, the average recall, precision, and F1 score for both validation and testing data are 94% and 95%, respectively. The findings of the authors indicate that the CNN model effectively detects true positive labels with a high degree of accuracy.

Coelho et al. [16] introduced an IoT system that has the ability to monitor water distribution systems and accurately detect and locate water leaks. The proposed solution uses flow sensors and affordable microcontrollers (ESP32) to collect and process real-time data. Five different classification algorithms were considered, namely, random forests, decision trees, neural networks, support vector machines, and XGBoost. A total of 12 tests were performed for each method in order to find the algorithm with the best accuracy for implementation of the system. The random forest algorithm consistently achieved the highest accuracy across various scenarios, making it the preferred choice with an accuracy of nearly 85%.

Loukatos et al. [17] presented a system that solves the issues of traditional IoT systems. Using embedded ML on a Raspberry Pi Pico microcontroller board, the authors trained a neural network to recognize three characteristic kinds of water utilization profiles which are Normal Use (NU), Water Leak (WL), and Water Waste (WW). The neural network structure has an input layer with 200 features (window size), two hidden layers, with the first one to have 20 neurons and the second one 10 neurons, and an output layer with three classes. Upon evaluating the testing data, the system achieved an accuracy of 77.8% for the NU category, indicating that it correctly identified Normal Use instances. Similarly, it achieved a 100% success rate for both the WW and WL categories, accurately identifying Water Waste and Water Leak scenarios. These performance results led to an expected accuracy of 98.5% for the final model when tested using the quantized (int8) version of the dataset.

The investigation of domestic water leak detection has also been examined through the analysis of flow data [18]. This research has focused on training a random forest and a CNN-based model in the cloud. The classification problem addressed in this study revolves around detecting leak events from non-leak events. They also detect the magnitude of leaks categorized as small (≤1 L/h), medium-sized (1 to 10 L/h), or large (≥10 L/h). The CNN model exhibited the best performance with an accuracy, precision, and recall ranging from 92% to 96%. Additionally, the area under the Precision-Recall (PR) and Receiver Operating Characteristic (ROC) curves consistently achieved high values, ranging between 97% and 99%. A summary of the salient features from the literature review is provided by Table 1.

The existing literature in the area of water leakage detection systems reveals a noticeable gap in the integration of TinyML methodologies. While prior efforts have been dedicated to tackling the water leakage challenge, a significant portion of these solutions has not capitalized on the potential benefits of TinyML approaches. Furthermore, the few endeavors that do involve TinyML techniques often exhibit limitations, particularly in terms of the scope and the considered CNN models. We make a contribution by experimenting with state-of-the-art CNN models, such as ResNets [19], MobileNet [20] and EfficientNet [21] architectures, for highly accurate and efficient water leakage detection. Moreover, a distinctive aspect of our work is the attention we devote to the integration of these models into small-scale devices using TinyML. This two-fold approach not only broadens the spectrum of the considered ML models but also paves the way for the seamless incorporation of cutting-edge technology into an efficient real-time operational framework of water leakage detection.

## 3. Materials and Methods

**Figure 1:**
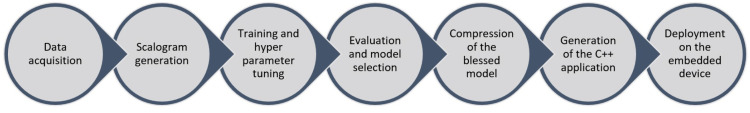
The systematic approach undertaken to achieve this study.

In this section, we detail the methodologies employed for the acquisition, preprocessing, and analysis of the data central to our study. Figure 1 provides a comprehensive visual representation of the approach we undertook to achieve this study. The process involves several key stages, similar to traditional machine learning projects, with some additional considerations for the constrained real-time processing requirements of the embedded device. We preprocess the acoustic leakage data and generate scalogram images. The different CNN models are trained and optimized. Hyperparameters are tuned on the validation set, and we perform evaluation using a separate testing test. The chosen model with the best testing metrics is optimized for on-device resource efficiency. In our research, we compressed the model using quantization and evaluated the model’s performance taking into account the device’s real-time hardware and real time constraints. Finally, we generate the C++ packages to be deployed on the device. The development lifecycle was conducted on Edge Impulse (https://edgeimpulse.com/ (accessed on 15 March 2023)), which is a platform that facilitates training models, tuning hyperparameters, as well as optimizing the models to run on any edge device.

### 3.1. Data Acquisition and Preprocessing

**Figure 2:**
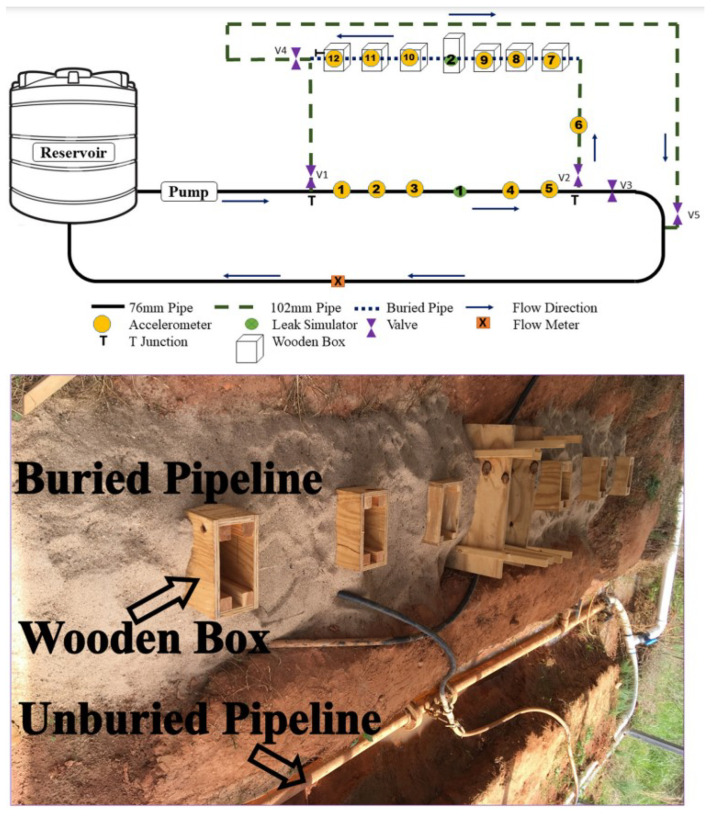
Schematic layout of the experimental pipeline setup (reprinted, with permission, from [14] @ 2020 Elsevier).

In this work, we use an existing dataset curated by Shukla et al. [14], collected using a small-scale experimental setup, that was built in the Clemson University campus (US), to collect data on pipeline vibrations. This system made use of accelerometers positioned at various locations throughout the pipe’s length. The test set included elements that mimic real-world complexity, including pipes with various diameters, rounded bends, T-joints, pipes at various heights, and various burial conditions. As shown in Figure 2, acceleration data were gathered using Bruel and Kjaer (BK) 4507-B-006 accelerometers from a total of 12 locations dispersed throughout the pipeline system. The test setup included two PVC pipes with diameters of 3 inches (76 mm) and 4 inches (102 mm). The 4-inch (102 mm) PVC pipeline was only partially covered (shown in Figure 2 by a dotted line); the rest of the system was left exposed. To make it easier to access the pipe surface and mount the accelerometers for data collection, wooden boxes were erected on the subterranean. Within the experimental test bed, the accelerometers were located at an average distance of 30 inches (762 mm) for the unburied pipeline and 24 inches (610 mm) for the buried pipeline. The test setup also comprised two leak simulators, one mounted on a PVC pipe with a diameter of 3 inches (76 mm) and the other on a pipe with a diameter of 4 inches (102 mm). More detailed information about them and the test setup can be found in [22,23].

As can be seen from Figure 2, accelerometers 1, 2, 3, 4, 5, and 6 detect whether a leak occurred in the leak simulator number 1, which is located in the unburied section. Similarly, accelerometers 7, 8, 9, 10, 11, and 12 detect leaks in the leak simulator number 2, which is located in the buried section. Table 2 shows the recorded flow information for all 25 situations that were acquired from the flowmeter. Each scenario’s acceleration signal data was recorded once the pipeline system reached a stable flow condition, as shown by repeatable flow-meter readings. Ten samples of 15-s data were taken for each scenario in order to guarantee precise readings and remove inadvertent biases or inaccuracies. To diversify the dataset, the size of leaks and the water flow are changed in each scenario [14].

Our research focuses on detecting whether a pipeline is leaky or not; thus, we processed the data in such a way it aligns with our objective by categorizing the data into two classes which are ‘Leak’ and ‘NonLeak’.

In order to make use of CNN models in the water leak context, the acoustic data should be converted into scalogram images. A scalogram is a representation of the continuous wavelet transform’s (CWT) absolute value that demonstrates how a signal varies with frequency and time [24]. In contrast, the fast Fourier transform (FFT) of the signal, which splits it into smaller parts, yields a spectrogram. Scalograms break down the signal into wavelets, whereas spectrograms break it down into infinite-duration sinusoids. The signal features represented in scalogram images are defined by wavelet parameters. Wavelets are useful for detecting sudden shifts in the signal by localizing it in both frequency and time, while scalograms are appropriate for studying low-frequency acceleration signals [24].

We were able to generate 1440 ‘NonLeak’ scalograms and 1680 ‘Leak’ scalograms, resulting in a total of 3120 scalograms. We have successfully achieved the crucial task of balancing the dataset by using data augmentation techniques, namely random cropping and noise injection. Random cropping involves randomly cropping a portion of the scalogram images during training; it helps the model become more robust to variations. As for noise injection, it makes the model more robust by simulating real-world conditions where data is often corrupted by various forms of noise. This transformative process has not only mitigated class imbalance but has also enhanced the generalization of our machine learning models by reducing overfitting.

**Figure 3:**
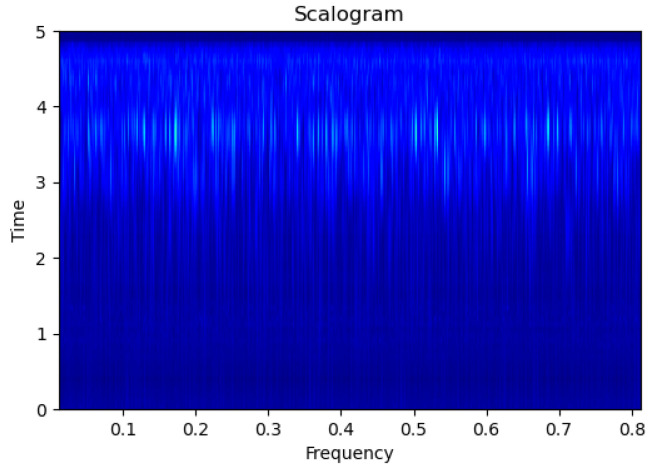
NonLeak|Unburied|Scalogram 1.

**Figure 4:**
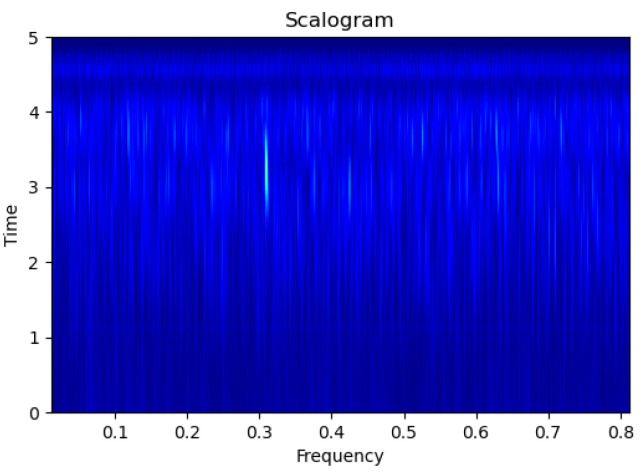
NonLeak|Unburied|Scalogram 7.

**Figure 5:**
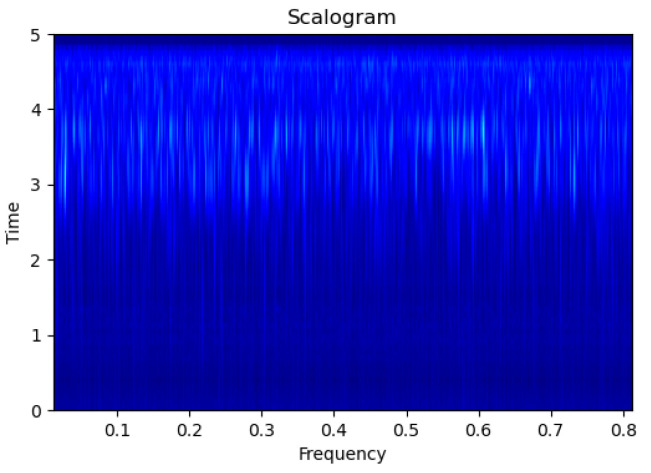
NonLeak|Unburied|Scalogram 18.

**Figure 6:**
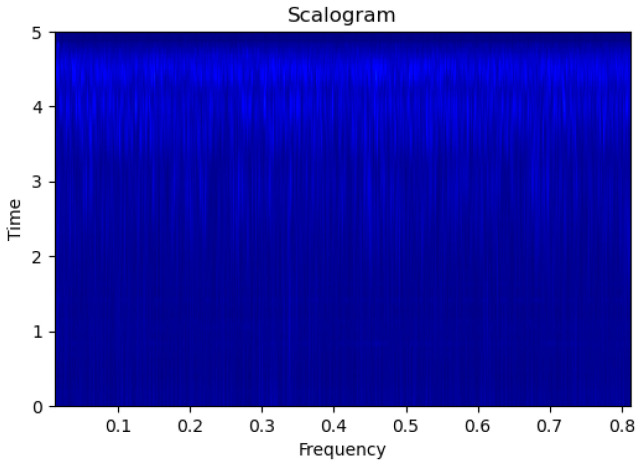
NonLeak|Buried|Scalogram 1.

**Figure 7:**
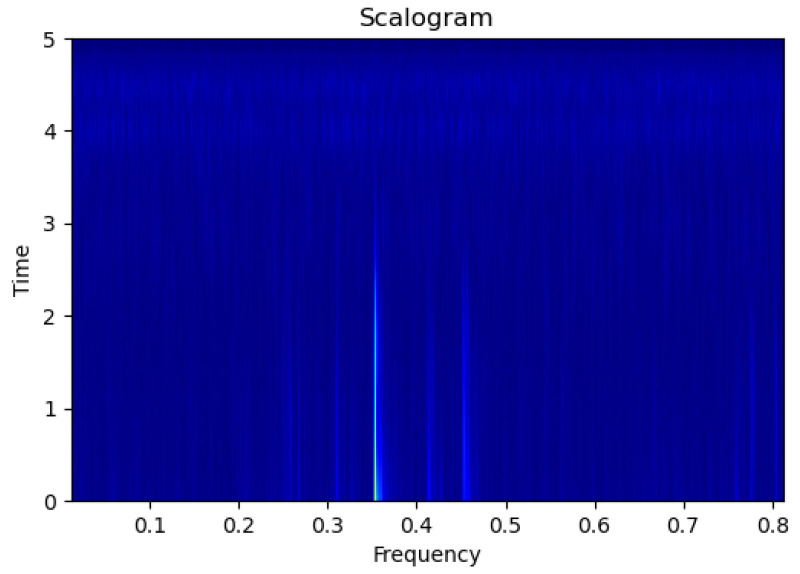
NonLeak|Buried|Scalogram 23.

**Figure 8:**
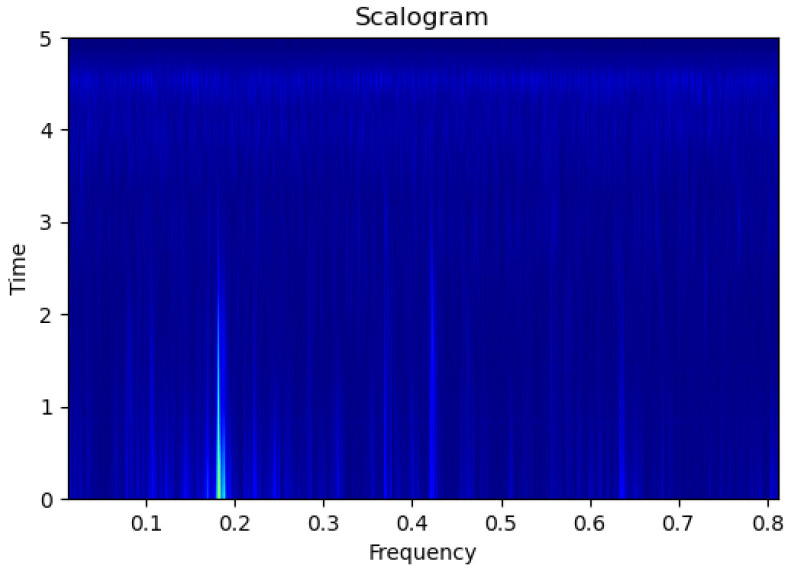
NonLeak|Buried|Scalogram 25.

**Figure 9:**
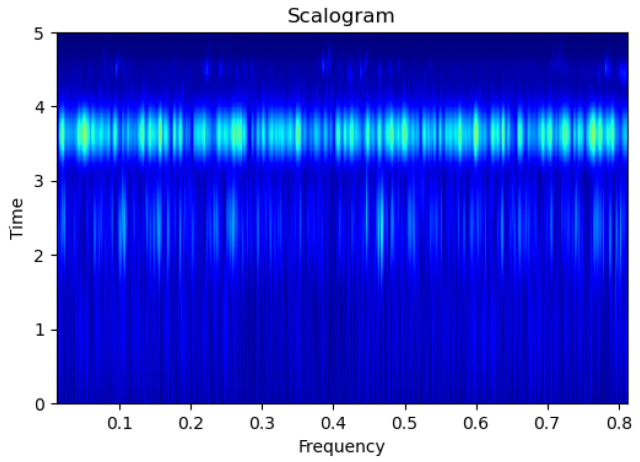
Leak|Unburied|Scalogram 5.

**Figure 10:**
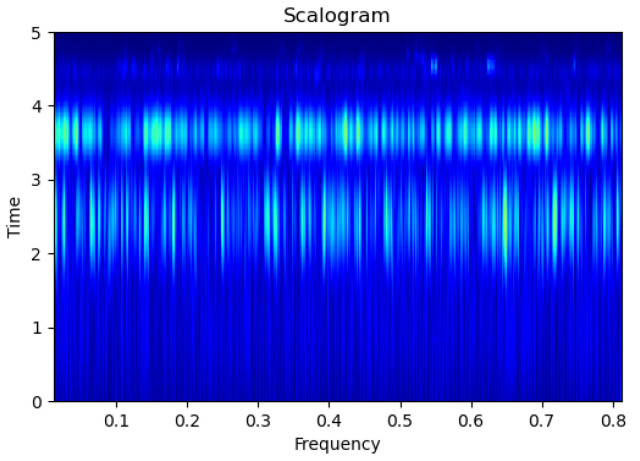
Leak|Unburied|Scalogram 17.

**Figure 11:**
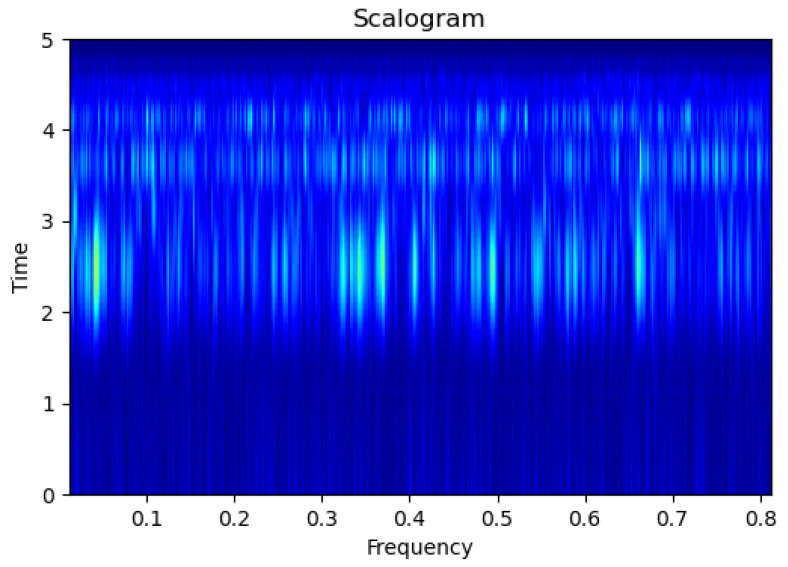
Leak|Unburied|Scalogram 23.

**Figure 12:**
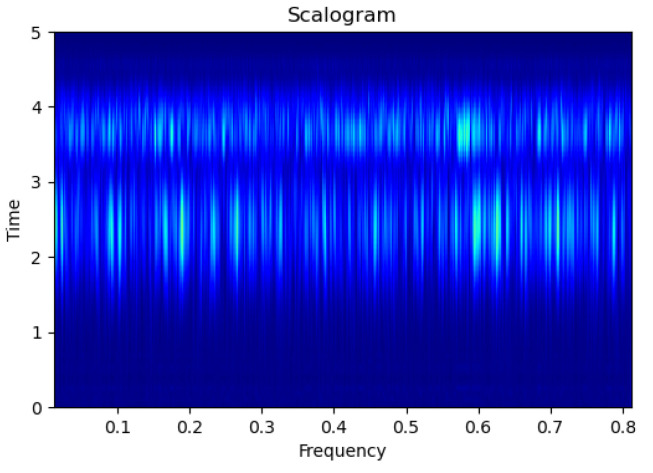
Leak|Buried|Scalogram 6.

**Figure 13:**
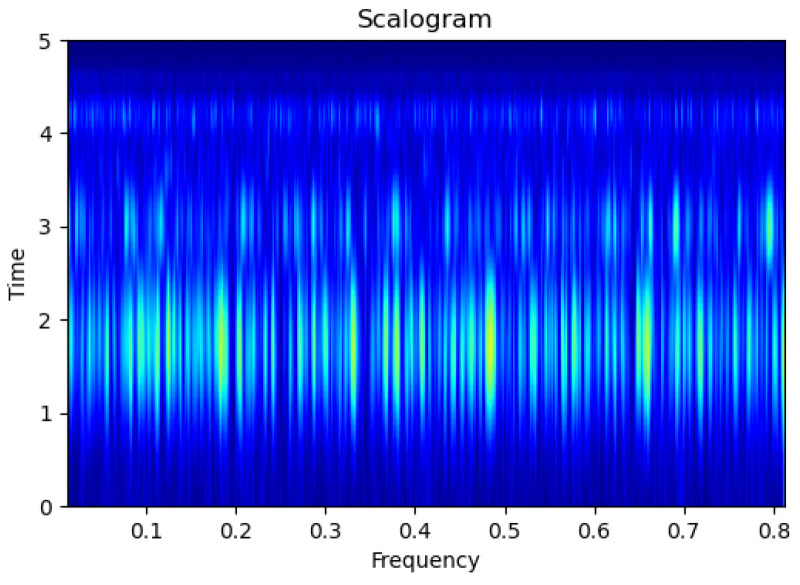
Leak|Buried|Scalogram 12.

**Figure 14:**
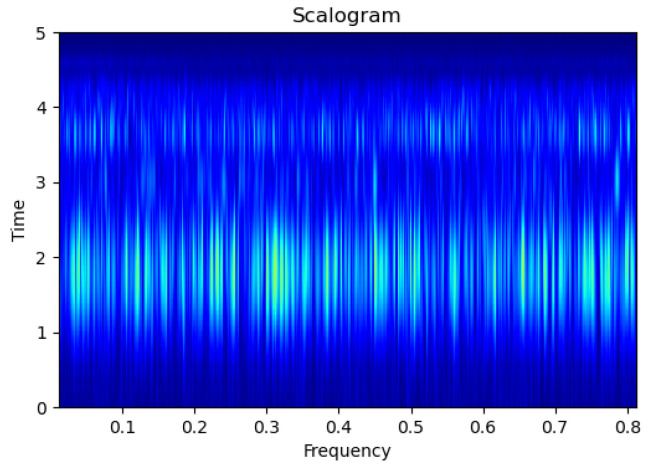
Leak|Buried|Scalogram 19.

Figure 3, Figure 4, Figure 5, Figure 6, Figure 7, Figure 8, Figure 9, Figure 10, Figure 11, Figure 12, Figure 13 and Figure 14 display the scalogram images of different scenarios of Leak, NonLeak, buried and unburied sections of the pipeline setup. Comparing the images, it is evident that the scalogram images of the buried section exhibit less noise and clearer features (indicated by heavy green spots) compared to the scalogram images of the unburied section. However, differentiating between scalogram images corresponding to non-leaky (Figure 3, Figure 4, Figure 5, Figure 6, Figure 7 and Figure 8) and leaky (Figure 9, Figure 10, Figure 11, Figure 12, Figure 13 and Figure 14) is challenging.

### 3.2. Water Leak Detection CNN Models

In this work, we used transfer learning to train several CNN models, namely AlexNet, ResNet, EfficientNet, MobileNet V1, and MobileNet V2. Each of these models possess distinct architectural characteristics and computational efficiencies, making them valuable candidates for our research.

AlexNet, developed by Krizhevsky et al. in 2012 [15], is a deep CNN model that played a pivotal role in revolutionizing image classification tasks. It consists of multiple convolutional layers, followed by fully connected layers, and employs techniques like ReLU activation, dropout regularization, and overlapping pooling. AlexNet demonstrated exceptional performance and significantly outperformed other existing models at the time of its introduction in spite of its relatively small size (61 million parameters).

ResNet, short for Residual Network, introduced by He et al. in 2015 [19], addressed the challenge of training extremely deep neural networks. It introduced the concept of residual connections, allowing the network to learn residual mappings and effectively tackle the vanishing gradient problem. By utilizing skip connections, ResNet enabled the training of deeper networks with improved accuracy and ease of optimization.

EfficientNet [21] aims to optimize both model accuracy and computational efficiency. The model’s architecture was obtained using a neural architecture search to find a good trade-off between model size and performance, making it highly efficient for resource-constrained environments. EfficientNet also employs a compound scaling method to systematically balance the depth, width, and resolution of the network. These features make EfficientNet transfer well to achieve state-of-the-art accuracy on several benchmark datasets, thus, it has become increasingly popular due to its ability to achieve impressive results with limited computational budgets.

MobileNets, introduced by Howard et al. in 2017 [20], focus on efficient mobile applications and low-power devices. MobileNets make use of depthwise separable convolutions to reduce the computational cost while maintaining reasonable accuracy. MobileNet architectures are well-suited for resource-constrained environments, enabling real-time image classification on devices with limited processing capabilities. In this research, we used two versions from this family of models, namely MobileNet V1 [20] and MobileNet V2 [25].

All five models have been pre-trained on the ImageNet dataset—a large-scale collection of approximately 1.2 million labeled images spanning 1000 different categories—to ensure a strong foundation in visual recognition and feature extraction. This extensive pre-training process equips the models with a rich understanding of a wide range of objects, scenes, and concepts, making them valuable tools for a variety of computer vision tasks.

These model choices reflect a balance between model complexity, resource efficiency, and historical significance, tailored to the specific requirements and constraints of the water leak detection project. Moreover, these models have demonstrated their effectiveness not only in traditional image classification but also in the context of scalogram images.

More details about the architectures of the used models can be found in Table 3.

We employed transfer learning techniques to leverage pre-existing knowledge in various domains and transfer it to our leak detection problem by capturing intricate patterns and representations in the water leak detection dataset. In essence, transfer learning reduces the time and computational resources required for model development. Considering that our dataset is small, as a transfer learning strategy, we froze the downloaded parameters and we trained only the parameters of the output layer. By strategically freezing the early layers of the neural network during training, we ensure that the weights and parameters in those layers remained unchanged, allowing the model to retain the knowledge it had acquired during its initial training phases.

We used ADAM as optimization algorithm, a technique that seamlessly combines two crucial optimization strategies [26]: Nesterov momentum, which aids in achieving better convergence, and adaptive learning rates which help adapting the learning rate for each parameter. We further employed a mini-batch gradient descent approach to make a good estimation of the error and the gradients. Our training strategy involved a specific number of epochs, which were determined by the tuner based on the optimal validation results, and this was around 50 epochs. To avoid overfitting, the dropout technique is used by the different models we applied transfer learning on. Furthermore, we used data augmentation which usually enhances generalization.

To tune the hyperparameters, a total of 15 combinations of the number of epochs, learning rate, and mini-batch size were tested over all five models. The details of the different combinations are listed in Table 4. The goal was to find the optimal hyperparameters that can improve the training time while improving the classification accuracy.

We evaluated the five models and compared the results. The performance of the models is evaluated based on accuracy, recall, precision and F1 score metrics [27,28]. The considered metrics offer a comprehensive assessment of model performance, catering to different aspects of prediction quality. Accuracy provides a holistic view of correct classifications, making it a good starting point. However, in many real-world scenarios, imbalanced datasets or specific business needs require a deeper analysis. Recall is crucial for identifying the proportion of true leak detection alerts among actual positives, emphasizing the model’s ability to catch relevant events. Precision complements recall by measuring the proportion of detected leaks among predicted positives, focusing on minimizing false positives. The F1 score strikes a balance between precision and recall, making it a reliable metric when striving for a harmonious trade-off between minimizing false positives and false negatives. These metrics together ensure a more nuanced evaluation and guide model refinement tailored to specific project objectives and constraints.

### 3.3. Target Deployment

The target device for deployment is an Arduino Nano 33 BLE Sense (Cortex-M4F 64 MHz, Arduino, Somerville, MA, USA), which is characterized by its compact size and suitability for various applications. With a maximum available RAM of 256 kB and a ROM of 1024 kB, the device offers limited computational resources. The Arduino Nano 33 BLE is equipped with five sensors that facilitate diverse data collection capabilities. Particularly relevant to our study is the acoustic data sensor. The onboard MEMS microphone enables the acquisition of acoustic signals, allowing for sound analysis and recognition tasks. Additionally, the integrated digital barometric sensor aids in capturing atmospheric pressure variations, which can be essential for understanding fluid dynamics and airflow patterns.

State-of-the-art CNN models, while powerful, are often too large to be deployed on a compact device, such as an Arduino Nano 33 BLE Sense, due to their resource-intensive nature. To address this challenge, we use post-training quantization offered by the Edge Impulse platform, a feature to optimize models based on implementations provided by the Tensorflow Lite Micro library. This approach involves strategically reducing the precision of a model’s internal representations by converting 32-bit floating-point parameters into lower precision int8. This helped us to significantly reduce the amount of memory (ROM) the model requires, making it much more feasible for deployment on a tiny device. Additionally, this reduction in memory usage results in faster computation, enabling quicker predictions and responses. In essence, post-training quantization serves as a crucial optimization step that allows us to retain the core functionality and accuracy of the models while tailoring them to the constraints of the device’s limited resources.

Considered that the water leak detection models are to be deployed in Arduino Nano 33 BLE Sense, other metrics were taken into consideration, namely the inference time which is how quickly the models can make predictions, the model’s memory usage and storage footprint. These factors are crucial because we want to make sure that the models are efficient enough and can fit and perform smoothly on an Arduino Nano 33 BLE. By examining their classification metrics, computational efficiency, and suitability for the tiny device, we were able to make informed decisions regarding the model selection.

To deploy the ML model on the Arduino Nano 33 BLE, we convert our application into a fully optimized C++ source code that can be integrated as an application on the device. The customizable library packages contain both the preprocessing block that turns acoustic data into a scalogram, and the machine learning block for inference; besides all of the external required libraries, everything is turned into a single package with all available source code.

## 4. Results and Discussion

In order to train and validate the CNN models, we used a split of 72%-10%-18% for training, validation and testing respectively. A 5-fold cross-validation approach is adopted where different sets of training and validation were randomly selected in each fold. Through training and evaluation cycles, it became evident that this split guaranteed a balance between providing the model with a enough and diverse training data and ensuring a robust assessment of its generalization performance. Table 5 shows the results of our experiments.

As Table 5 shows, EfficientNet emerged as the model that showed the best results. The reasons for this outstanding performance were numerous. First of all, EfficientNet’s design consists of a revolutionary compound scaling technique that uniformly scales the depth, width, and resolution dimensions, allowing it to outperform other models. Due to this quality, EfficientNet was able to balance model size and accuracy, providing superior performance. Furthermore, extensive pre-training on massive datasets helps EfficientNet learn rich and generalized representations. Its effective network block designs and depth-wise separable convolutions aid in lowering computational costs while keeping expressive capability. As a result, EfficientNet’s innovative architectural design and enhanced efficiency features were crucial in providing maximum performance for our research. ResNet comes second for several reasons. ResNet’s deep residual connections allow for effective propagation of gradients, addressing the issue of vanishing gradients and enabling the network to be trained more deeply. The skip connections in ResNet facilitate the flow of information, helping the model capture more complex patterns and features. With a deeper architecture compared to MobileNet and AlexNet, ResNet has a greater capacity to learn intricate representations, which can lead to improved performance. Although ResNet’s computational complexity is higher than EfficientNet and MobileNet, its architectural design and ability to handle deeper networks contribute to its notable performance, placing it in the second position for accuracy in our study. However, this emphasis on depth alone does not necessarily guarantee optimal performance. The deeper architecture of ResNet also leads to higher computational complexity, which may limit its practicality in certain resource-constrained scenarios.

In light of the constraint of the device considered for deployment, our research has led us to compress the five models using quantization; however, two of them, namely EfficientNet and MobileNet V1, fit within the device characteristics range. Table 6 shows the inference time, ram and flash usage of the developed models.

Among the various models explored, EfficientNet emerged as the optimal choice, effectively striking a balance between good performance and compatibility with our tiny device by adhering to its stringent resource limitations.

## 5. Conclusions

This study highlights the significance of developing advanced techniques for leak detection, especially in the context of water distribution networks and buildings’ pipelines. The integration of deep learning algorithms, such as the proposed approach utilizing scalogram images of vibration signals, shows promise in efficiently identifying and locating leaks. By leveraging TinyML, which combines sensor technology and ML at the network edges, real-time data collection, analysis, and localized decision-making can be achieved. This not only enhances the accuracy and efficiency of leak detection but also reduces reliance on centralized entities.

The experimental results show that EfficientNet underscores our commitment to not only achieving noteworthy performance but also to ensuring seamless integration into the tiny device considered for deployment. Its capacity to achieve good performance with resource-efficient design positions it as the ideal candidate for our deployment objectives, reaffirming our endeavor to harness cutting-edge technology for practical applications on constrained hardware.

Moreover, the high recall achieved by the model also guarantees a reduced number of false alarms. The edge solution can process data locally and filter out noise or false alarms before sending alerts, reducing the likelihood of unnecessary responses.

This research has culminated in the development of a water leak detection embedded model. As we move forward, our focus will shift towards the practical implementation of a water leak detection solution in domestic smart buildings. This step entails the translation of our findings to seamlessly integrate the embedded ML system into a real-world environment with hardware and environmental constraints. The deployment process will encompass rigorous testing, optimization, and fine-tuning to ensure reliability and effectiveness.

## Figures and Tables

**Table 1:** Related work summary.

Research Paper	Data/Sensors	Application Domain	Model Deployment	Classification Problem	Model(s)	Results	
[11]	Pressure data, JOHNSON CONTROLS (P499ABS-401) sensor	Water distribution systems	No specific target	13 categories depending on leakage point and position	CNN model	Accuracy	97.33%
[12]	Accelerometer data, piezoelectric accelerometers (PCB-393B31)	Water distribution systems	Backend server	Leak, non-leak conditions	Ensemble 1D-CNN-SVM model, graph-based localization algorithm	Accuracy Sensitivity Specificity AUC	99.3%, 98.2%, 99.8%, 99.9%
[13]	Acoustic signals, hydrophone sensor	Water distribution networks	No specific target	Leak, non-leak conditions	CNN with VAE (Variational autoencoders)	Accuracy Precision Recall	97.2%, 92%, 96%
[14]	Accelerometer data (available upon request from authors)	Infrastructure monitoring	Offline sensitivity analysis–not applicable	25 scenarios depending on leakage flow	CNN adapted from AlexNet	Average Accuracy	94–98% depending on locations
[16]	Water flow data	Agriculture	IoT, cloud	No leaks, micro, minor, major leaks	Random Forest	Accuracy	85%
[17]	Water flow data	Smart Agriculture	Edge	Normal Use, Water Leak, Water Waste	Multi-layer Perceptron Neural Network	Accuracy	98.5%
[18]	Water flow data	Domestic water	Cloud	No leaks, small, medium and large leaks	CNN	Accuracy Precision Recall	92–96%

**Table 2:** The water leakage data scenarios (reprinted, with permission, from [14] @ 2020 Elsevier).

Scenarios	Leak 1 (GPM)	Leak 2 (GPM)	Flow Rate (GPM)
SC1	0	0	163
SC2	1	0	160.6
SC3	1	1	159.2
SC4	5.28	1	157.7
SC5	14.73	1	156.8
SC6	22.63	1	152.8
SC7	0	1	163.5
SC8	0	5.19	160
SC9	1.04	5.19	159
SC10	1.04	14.08	156
SC11	1.04	23.46	154
SC12	0	23.46	153
SC13	4.73	23.46	152
SC14	13.77	23.46	148
SC15	22.63	23.46	143
SC16	22.63	4.53	150.9
SC17	22.63	13.77	146
SC18	0	13.77	156
SC19	14.4	13.77	149
SC20	14.4	5.66	152
SC21	4.73	5.66	156
SC22	4.73	13.77	152
SC23	4.73	0	158.8
SC24	13.77	0	157
SC25	21.12	0	155

**Table 3:** Models’ architecture.

Model	Architecture
AlexNet [15]	Convolutional layers with kernel sizes (11 × 11, 5 × 5, 3 × 3), ReLU activation, and overlapping max pooling, followed by fully connected layers with dropout and softmax activation for classification.
ResNet [19]	Residual blocks with shortcut connections. Each block consists of multiple convolutional layers with batch normalization and ReLU activation. Global average pooling is applied followed by fully connected layers with softmax activation for classification.
EfficientNet [21]	Compound scaling technique that scales the depth, width, and resolution of the network. It consists of convolutional layers with efficient bottleneck structures, swish activation, and batch normalization. Global average pooling is applied followed by fully connected layers with softmax activation for classification.
MobileNet V1 [20]	Depthwise separable convolutional layers, which split the standard convolution into separate depthwise and pointwise convolutions. It includes ReLU activation, batch normalization, and depthwise and pointwise convolutional layers. Max pooling is applied, followed by fully connected layers with softmax activation for classification.
MobileNet V2 [25]	Inverted residual blocks with linear bottlenecks. It employs depthwise separable convolutions, skip connections, and expansion layers to improve efficiency. The architecture also incorporates ReLU6 activation, batch normalization, and global average pooling. Fully connected layers with softmax activation are used for classification.

**Table 4:** Combinations of hyperparameters.

Cases	Epochs	Learning Rate	Mini-Batch Size
Case 1	10	5 × $10^{-3}$	8
Case 2	25	5 × $10^{-3}$	8
Case 3	50	5 × $10^{-3}$	8
Case 4	10	5 × $10^{-4}$	8
Case 5	25	5 × $10^{-4}$	8
Case 6	50	5 × $10^{-4}$	8
Case 7	10	5 × $10^{-4}$	16
Case 8	25	5 × $10^{-4}$	16
Case 9	50	5 × $10^{-4}$	16
Case 10	10	5 × $10^{-4}$	32
Case 11	25	5 × $10^{-4}$	32
Case 12 *	50	5 × $10^{-4}$	32
Case 13	10	5 × $10^{-5}$	32
Case 14	25	5 × $10^{-5}$	32
Case 15	50	5 × $10^{-5}$	32

* Case showing highest accuraccies.

**Table 5:** Models’ evaluation on validation and testing sets (The best results were made bold).

Model	Accuracy	Precision	Recall	F1 Score
**AlexNet**				
validation	98.00%	99.10%	96.97%	98.02%
testing	96.24%	96.70%	95.46%	96.08%
**ResNet**				
validation	99.20%	100.0%	98.43%	99.21%
testing	96.76%	96.70%	96.89%	96.80%
**EfficientNet**				
validation	99.55%	99.10%	100.0%	99.55%
testing	**97.45%**	96.70%	**98.57%**	**97.63%**
**MobileNet V1**				
validation	98.65%	99.00%	98.51%	98.75%
testing	96.24%	96.70%	95.46%	96.08%
**MobileNet V2**				
validation	99.25%	100.0%	98.52%	99.26%
testing	96.76 %	96.70%	96.89%	96.80%

**Table 6:** Models’ inference time, peak ram and flash usages.

Model	Inference_Time (MS)	Peak Ram Usage (KB)	Flash Usage (KB)
AlexNet	20,843	132.4	20.7 × 10${}^{3}$
ResNet	1958	333.8	640.7
EfficientNet	1932	255.3	48.7
MobileNet V1	3156	253.5	310.8
MobileNet V2	5200	720.8	580.2

## Data Availability

Data subject to third party restrictions: The data that support the findings of this study are available from Shukla et al. [14] and are available from the authors with the permission of Shukla et al. [14].
